# Unraveling the Gut Microbiome of the Invasive Small Indian Mongoose (*Urva auropunctata*) in the Caribbean

**DOI:** 10.3390/microorganisms9030465

**Published:** 2021-02-24

**Authors:** Anne A. M. J. Becker, KC Hill, Patrick Butaye

**Affiliations:** 1 Department of Biomedical Sciences, Ross University School of Veterinary Medicine, Basseterre, Saint Kitts and Nevis; kchill8@gmail.com (K.H.); pbutaye@rossvet.edu.kn (P.B.); 2 One Health Center for Zoonoses and Tropical Veterinary Medicine, Ross University School of Veterinary Medicine, Basseterre, Saint Kitts and Nevis; 3 Department of Pathology, Bacteriology, and Poultry Diseases, Faculty of Veterinary Medicine, Ghent University, 9820 Merelbeke, Belgium

**Keywords:** small Indian mongoose, microbial profiling, gut microbiota, Caribbean, invasive species, *Herpestes*, *Urva auropunctata*, carnivore

## Abstract

Small Indian mongooses (*Urva auropunctata*) are among the most pervasive predators to disrupt the native ecology on Caribbean islands and are strongly entrenched in their areas of introduction. Few studies, however, have considered the microbial ecology of such biological invasions. In this study, we investigated the gut microbiota of invasive small Indian mongooses in terms of taxonomic diversity and functional potential. To this end, we collected fecal samples from 60 free-roaming mongooses trapped in different vegetation zones on the island Saint Kitts. The core gut microbiome, assessed by 16S rRNA amplicon gene sequencing on the Ion S5^TM^ XL platform, reflects a carnivore-like signature with a dominant abundance of Firmicutes (54.96%), followed by Proteobacteria (13.98%) and Fusobacteria (12.39%), and a relatively minor contribution of Actinobacteria (10.4%) and Bacteroidetes (6.40%). Mongooses trapped at coastal sites exhibited a higher relative abundance of *Fusobacterium* spp. whereas those trapped in scrubland areas were enriched in Bacteroidetes, but there was no site-specific difference in predicted metabolic properties. Between males and females, beta-diversity was not significantly different and no sex-specific strategies for energy production were observed. However, the relative abundance of Gammaproteobacteria, and more specifically, *Enterobacteriaceae*, was significantly higher in males. This first description of the microbial profile of small Indian mongooses provides new insights into their bioecology and can serve as a springboard to further elucidating this invasive predator’s impact throughout the Caribbean.

## 1. Introduction

Studying different aspects of the bioecology of wild mammalian species is central to integrative wildlife conservation and habitat protection. The bioecology of a species encompasses different aspects, such as geographic range and habitat, feeding ecology, social behavior, genetics, morpho-physiology, and the host-associated gut microbiome which is more recently recognized as an integral part of a species’ bioecology [1]. This has led to increased microbiome research in rare, threatened, or valuable wildlife populations [2] while invasive species usually remain under the radar.

Listed as one of the world’s 100 worst invasive species [3] are small Indian mongooses (Carnivore, *Herpestidae*, *Urva auropunctata* (Hodgson, 1836), formerly *Herpestes auropunctatus* [4]), native to areas from the Middle East to Myanmar, but introduced for biological control to other regions worldwide between the late 19th and early 20th century [5]. The first introduction to the Caribbean islands for rodent control on sugarcane plantations dates back to 1870, with an introduction to the islands Saint Kitts and Nevis in 1884 [6]. Mongoose population density ranges now from 1 to +10 mongooses/ha in the Caribbean [5] and is estimated to be over 45,000 on St. Kitts. As the perception that mongooses are useful in rodent control weaned, they rapidly became a pest species, with damaging threats to native fauna, domestic poultry production, and public health [7,8]. Contributing to its immense success as an invasive species is its adaptability and high level of dietary flexibility. Small Indian mongooses are slender small-bodied predators (length 54–59 cm and body weight 434–650 g on average [5]) that occupy a wide range of habitats including open, scrubs, and dry and sub-tropical forests, but also coastal habitats, mangroves, and anthropized environments [9,10]. They are opportunistic generalists and feed on a variety of prey items, including rats, lizards, crustaceans, insects, seeds, birds, eggs, vegetable matter, carrion, and human refuse [8,11]. In recent years, their genetics [4], reproduction and feeding pattern [5,8,11] have been studied to some extent but the bioecology of small Indian mongooses remains to be unraveled in several dimensions, including the gut microbiota.

No study has actually investigated the gut microbial diversity of *Herpestes* species, except for one recent study describing the gut bacterial taxonomic and functional traits across sex and age classes in Egyptian mongooses (*Herpestes ichneumon*) in South Portugal. Compositional differences in gut microbiota were identified across sex and age classes but no significant difference in beta-diversity. Additionally, differential functional profiles between males and females disclosed potential sex-specific strategies to produce energy [12]. Egyptian mongooses belong to another phylogenetic clade than small Indian mongooses [13]. Moreover, they are medium-sized carnivores commonly found in southern Europe, with a markedly different feeding ecology and biogeography [14]. Both of these factors have been shown to profoundly shape gut microbiota in wild animals [15,16]. Consequently, caution is warranted with direct extrapolations of microbiome data between different *Herpestes* species.

The continued prevalence of the small Indian mongoose as an invasive species on Caribbean islands remains an important issue for ecosystem stability, protection of endemic species, and disease transmission. Therefore, we investigated the mongoose bioecology by molecular profiling of the bacterial diversity and functional potential of the small Indian mongoose gut microbiome.

## 2. Materials and Methods 

### 2.1. Sample Collection and Trapping Location

All samples were obtained between April and July 2017 from wild and free-roaming populations of small Indian mongooses located on the Caribbean island St. Kitts (17.357° N, 62.783° W) of the federation of St. Kitts and Nevis. Mongooses were trapped in live box traps (19 × 19 × 48 cm; Tomahawk Live Trap, Wisconsin) to which they were attracted by the presence of a bait consisting of tuna or chicken wings. Traps were set out in shaded areas at dawn and collected 4 to 5 h later. Trapped mongooses were immediately transported to the Necropsy Laboratory at Ross University School of Veterinary Medicine (RUSVM) and contact between animals was avoided at all times. Animals were subsequently anesthetized with 3 mL vaporized isoflurane and upon induction of the anesthetic state, the animal was removed from its cage and via a mask connected to an isoflurane vaporizer with controlled airflow of oxygen and isoflurane. Euthanasia followed via intra-cardiac injection of potassium chloride (1–2 mmol/kg) with a subsequent complete gross pathology examination and collection of different samples. Fecal samples were directly retrieved post-mortem from the rectum and distal part of the colon, aliquoted into sterile tubes and stored at −80 °C. For all mongooses, the body condition score (BCS) on a 5-range scale (1 = emaciated to 5 = obese) as well as any lesions, signs of inflammation, gestation, or pathological abnormalities were recorded. Mongooses were also allocated to one of four age classes defined by skull size and tooth-wear criteria [5]. These age classes encompassed juveniles, young adults, adults, and senior animals (Table S1).

Mongooses were trapped in the parishes Saint George Basseterre, Saint Peter Basseterre, and Saint Mary Cayon, which include urban, peri-urban, and rural areas with a quarry, a protected National Park (Royal Basseterre Valley Aquifer), and a variety of land vegetation ranging from coastal sands and rocks, grassy areas with sugar cane and minor crops, to drought deciduous scrubland [17]. Based on land use and vegetation cover, trap locations were grouped into nine different trapping areas for which detailed description is provided in Table S2.

This study was approved by the Ross University Institutional Animal Care and Use Committee under the IACUC Number 17.04.13. Trapping and necropsy of mongooses have been conducted according to all approved protocols within the period of approval from 04.04.2017 to 04.04.2020.

### 2.2. DNA Extraction, PCR, Library Preparation, and Sequencing

DNA extraction from 60 fecal samples (collected from 60 different mongooses) and 16S rRNA gene amplicon sequencing were performed using the Ion S5^TM^ XL sequencing platform, according to the manufacturer’s instructions. Briefly, fecal DNA was extracted using CTAB/SDS method [18] and included three DNA extraction blanks without the addition of any starting material except for ultrapure water. DNA concentrations of all samples were quantified on a NanoDrop 2000 spectrophotometer (Thermo Scientific) and samples were diluted to 1 ng/μL using sterile water before sending to Novogene Bioinfomatics Technology for PCR amplification with Phusion^®^ High-Fidelity PCR Master Mix (New England Biolabs). Barcoded primers (341F-806R) were used to target the V3–V4 hypervariable regions of the 16S rRNA gene. PCR products were detected on 2% agarose gel electrophoresis and samples with bright main band between 400–450 bp were further included in analyses. Amplicons were mixed in equidensity ratios and purified with GeneJET^TM^ Gel Extraction Kit (Thermo Scientific). Sequencing libraries were generated using Ion Plus Fragment Library Kit 48 rxns (Thermo Scientific) following manufacturer’s recommendations, quantified via Qubit, and sequenced on an Ion S5^TM^ XL platform with generation of 400 bp single-end reads.

### 2.3. Sequence Processing, Quality Control, OTU Clustering, and Species Annotation

Single-end reads were assigned to samples based on their unique barcode and truncated by cutting off the barcode and primer sequence using the Cutadapt quality controlled process [19]. All reads retrieved in the controls were removed from the samples. Quality filtering on the raw reads were performed under specific filtering conditions to obtain the high-quality clean reads according to the QIIME quality controlled process (v1.7.0, split_libraries_fastq.py script) [20]. Reads were eliminated whose low-quality nucleotides (Q-values ≤ 20) exceeded the threshold (40% of read length), which contained N nucleotides over the threshold set at 10% of the read length by default and which overlapped with the adapter over 15 bp threshold. Reads were also chimera filtered by aligning to the 128 Silva database using the UCHIME algorithm [21]. Data control software was ng-QC, developed by Novogene Bioinformatics Technology, with the threshold of low-quality base at 5. The percentage of clean reads in raw reads in the samples was on average 87% (Table S3). The high-quality reads were picked into distinct operational taxonomic units (OTUs) using Uparse pipeline (v7.0.1001) [22] with a 97% DNA sequence similarity threshold, and the Silva Database [23] was used based on the Mothur algorithm to annotate taxonomic information. Singletons were removed from analyses and only OTUs with at least 1% total abundance over all samples were retained. Multiple sequence alignments were conducted using the MUSCLE software (v3.8.31) [24] to study the phylogenetic relationship of different OTUs, and OTUs abundance information was normalized for subsequent analysis of alpha and beta diversity. Data are available under BioProject accession number PRJNA688145 and a Fasta file (Supplementary File 1) in the Supplementary Materials.

### 2.4. Data Analyses

Alpha diversity indices (Shannon Diversity Index, Observed Species, Chao 1, Simpson, ACE) were calculated in QIIME (v1.7.0) [25] and displayed with R software (v 2.15.3 and v 4.0.3) [26]. To assess sequencing depth and the current state of sampling, the Good’s coverage [27] was calculated and rarefaction and Specaccum curves constructed. Between-sample beta diversity was calculated in QIIME using both weighted and unweighted UniFrac distances based on species abundance and phylogenetic branch length. PCoA analysis was displayed by WGCNA (v 1.69) [28], ggplot2 (v 3.2) [29], and stats (v 3.6.2) packages in R. Statistical analyses in R tested the significance of community composition and structure differences between different sexes (Wilcoxon tests) and sampling locations (Anosim). To infer and predict the differential functional profile of the mongoose population, the PICRUSt genome prediction software was used [30]. OTUs were assigned at 97% similarity and mapped to the Greengenes v.13.5 database for functional prediction, with normalization to control for differences in 16S rDNA copy number among OTUs. Functional predictions were assigned up to KEGG Orthology (KO) level 2 for all genes. To simplify analyses, only second-level functions within the level 1 KEGG Orthologs “cellular processes”, “metabolism”, “genetic information processing”, and “environmental processing” were analyzed further, as the categories of “organismal systems” and “human disease” were thought to be poorly relevant to these samples. Spearman rank correlations relating second-level KO functional abundances and top 10 taxonomic phyla and family abundances were performed using R software.

## 3. Results

### 3.1. Sample Collection, Sequencing, and Quality Control

A total of 60 fecal samples were collected postmortem from 60 mongooses randomly trapped at 9 different areas on the Caribbean island St. Kitts. Upon routine postmortem examination of the mongooses (32 female and 28 male), the majority of the animals did not show pathologies except for some individuals with keratopathies or uterine, mammary, or subdermal masses. For one animal, extensive signs of enteritis with swollen mesentery lymph nodes was recorded. The majority of the mongooses belonged to age class 2 (young adult) or 3 (adult) and were well-conditioned with BCS 3. These classification criteria and metadata associated with the individuals are listed and described in more detail in Table S1. Fecal samples were analyzed by 16S rRNA gene sequencing and after filtering out low quality reads, a total of 8,740,709 reads were obtained with 145,678 ± 24,878 reads per sample on average. The average read length was 413 bp.

### 3.2. Gut Microbiota Profile of Small Indian Mongooses (Urva auropunctata)

**Figure 1:**
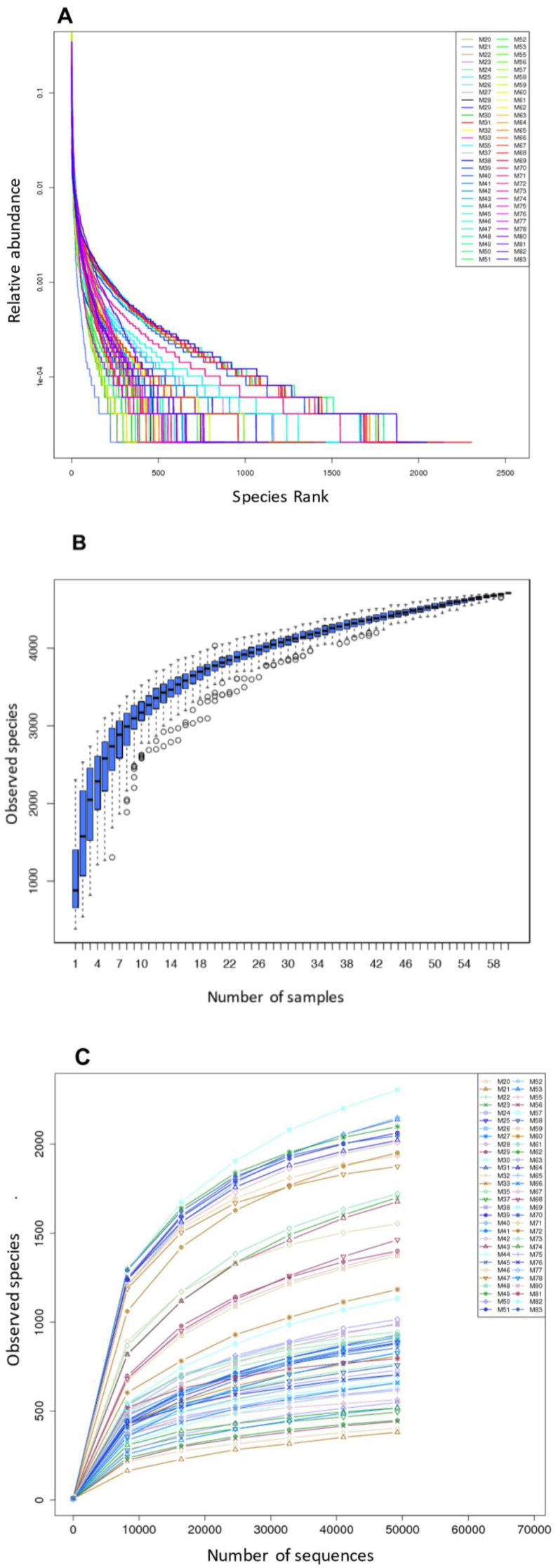
The rarefaction and extrapolation sampling curves based on the 16S rRNA gene sequencing data from 60 fecal samples of small Indian mongooses (sample codes: M_n_): (**A**) Rank-abundance distribution curve; (**B**) Specaccum curve; (**C**) Rarefaction curves.

After rarefaction to 49,228 reads/sample, high-quality reads were sorted into 4709 operational taxonomic units (OTUs) using a sequence identity cutoff of 97%. On average, we detected 1343 ± 576 OTUs per sample. The fecal bacterial distribution pattern was dominated by one to several genera and the rank abundance curves had a long tail of less abundant organisms (Figure 1A). The saturated Specaccum curve indicated that the sampling (60 samples) was comprehensive (Figure 1B). The shape of the rarefaction curves also evidenced a plateau (Figure 1C). Good’s coverage was >99% for sequences in each of the samples, indicating that the 16S rRNA gene sequences identified represented almost all the bacterial sequences present in the fecal samples. The alpha diversity of each sample was estimated by richness indices (observed species, Chao, and ACE (abundance-based coverage estimator) index) and the Shannon Diversity index, and is summarized in Table S4.

**Figure 2:**
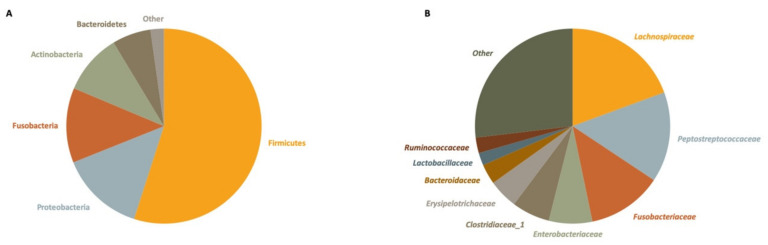
Relative abundance of the 10 most abundant bacterial phyla (**A**) and families (**B**) in 60 fecal samples of small Indian mongooses, determined by 16S rRNA gene sequencing.

Taxonomic assignment revealed a Firmicutes to Bacteroidetes ratio of 8.59, a dominant presence of Firmicutes (54.96% of total reads), Proteobacteria (13.98%) and Fusobacteria (12.39%), and a lower relative abundance of Actinobacteria (10.04%) and Bacteroidetes (6.40%). The remainder of reads could be attributed to marginal phyla (2.23%) (Figure 2A). The 10 most abundant families were each present in more than 86% of samples and together made up 44.25% of the total reads (Figure 2B). These comprised the *Lachnospiraceae* (19.38%), *Peptostreptococcaceae* (14.96%), *Fusobacteriaceae* (12.39%), *Enterobacteriaceae* (7.20%), *Clostridiaceae_1* (6.30%), *Erysipelotrichaceae* (4.88%), *Bacteroidaceae* (3.34%), *Lactobacillaceae* (2.09%), *Ruminococcaceae* (2.57%), and *Leuconostocaceae* (0.63%).

**Figure 3:**
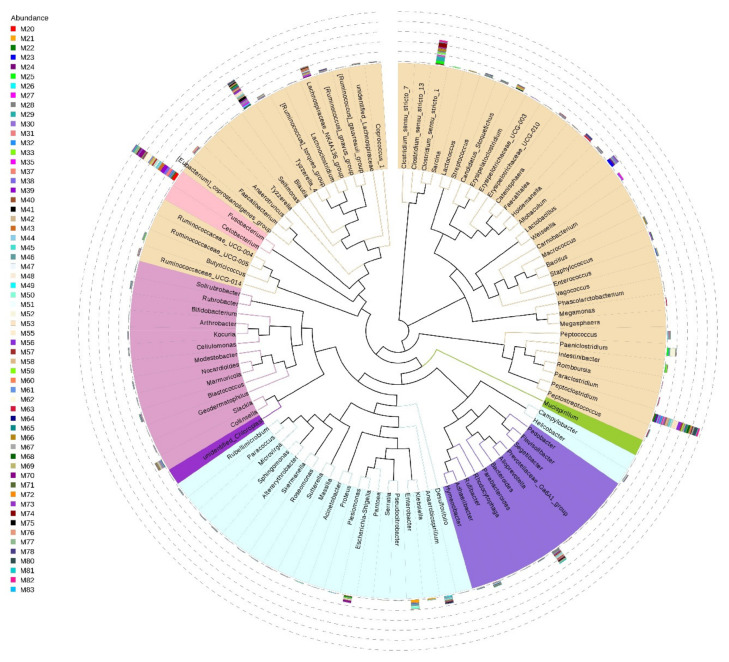
Phylogenetic evolutionary tree of the gut microbiota at genus level from small Indian mongooses. Different colors of the branches represent different phyla (pink: Fusobacteria, brown: Firmicutes, purple: Bacteroidetes, blue: Proteobacteria, dark pink: Actinobacteria, green: Deferribacteres). The relative abundance of each genus is displayed outside the circle with different colors representing different fecal samples from each animal that has a unique Mx number.

Among the 622 genera detected (80.15% of total reads), 400 (64.3%) were present in less than 50% of samples and only 50 (8%) were shared among samples. The genera holding >1.5% of the total dataset and represented in all samples are *Fusobacterium*, *Peptoclostridium*, *Blautia*, *Clostridium_sensu_stricto_1*, *Bacteroides*, and *Lachnospiraceae_NK4A136_group* (Figure 3).

### 3.3. Biogeography and Sex-Related Patterns of Gut Microbial Diversity in Small Indian Mongooses

**Figure 4:**
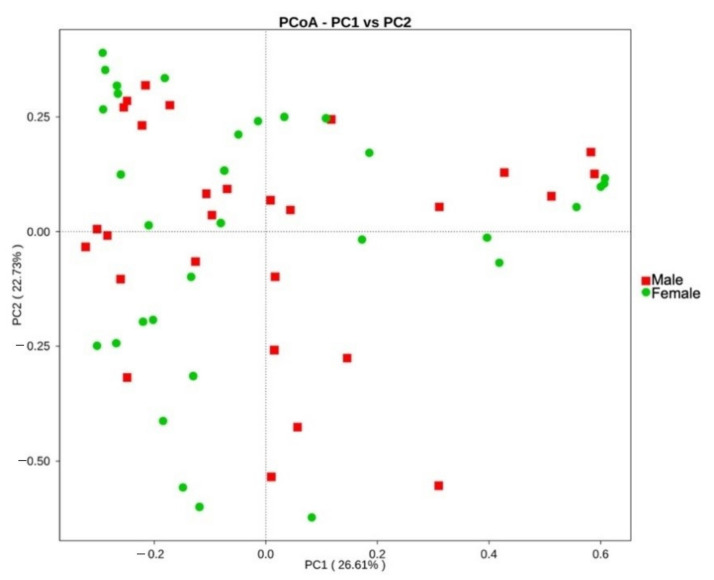
Principal component analysis (PCoA) of the gut microbiota of male (red, *n* = 28) and female (green, *n* = 32) small Indian mongooses, based on weighted UniFrac distances.

The fecal microbiota was further compared between age and BCS metrics, sexes, and different trapping locations on the island St. Kitts. No significant differences in diversity indices were observed between different age classes or BCS metrics. Overall, bacterial community structure and phylogenetic diversity did not significantly differ between sexes in the UniFrac unweighted beta diversity analysis, as distinct clusters could not be observed (*p* = 0.4287; Figure S1). However, the significance in the weighted analysis (*p* = 0.0498; Figure 4) suggests that similar taxa are present between sexes, but a difference in proportional abundance of the taxa occurs.

**Figure 5:**
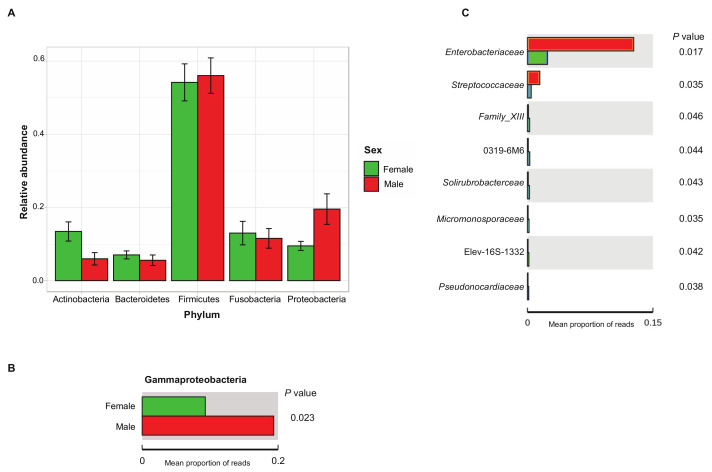
Relative abundance of the 5 most abundant bacterial phyla of each group (male, female) expressed as average ± SEM (**A**): the significantly different relative abundance of Gammaproteobacteria between male and female small Indian mongooses (*p* < 0.05, between-group Welch’s *t*-test analysis); (**B**,**C**): the eight discriminating genera significantly different in abundance between male and female small Indian mongooses (*p* < 0.05, between-group Welch’s *t*-test analysis).

Comparative analysis of OTUs across male and female samples evidenced that the majority of OTUs were shared across individuals, with 3435 common OTUs and only 764 unique to females and 510 to males. Comparison of the relative abundance of phyla between sexes reveals only a significantly higher abundance of Proteobacteria in males, more specifically, the Gammaproteobacteria (*p* < 0.05, between-group Welch’s *t*-test analysis; Figure 5A,B). At family level, we identified eight genera with significantly different abundance between sexes, of which *Enterobacteriaceae* had the highest discriminative power with a relative abundance five times higher in males than in females (*p* = 0.01, between-group Welch’s *t*-test analysis; Figure 5C).

**Figure 6:**
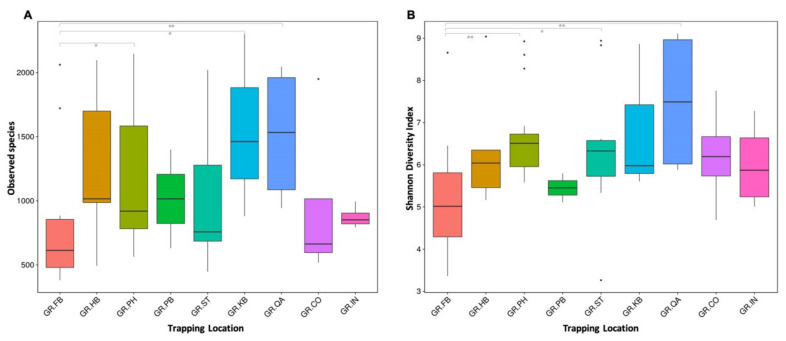
Boxplots based on Observed Species (**A**) and Shannon Diversity indices (**B**) showing the difference in alpha diversity between sampling locations. Significant differences between groups are marked with * (Wilcoxon, *p* < 0.05) and ** (Wilcoxon, *p* < 0.01) and solid dots represent outlier points.

Mongooses were trapped at nine different locations on the east side and mostly dense inhabited areas of the island St. Kitts. The land cover and forest formation for each area has been described in Supplementary Table S2 and is based on descriptions from satellite imagery provided by Helmer et al. [17]. Within-sample diversity (alpha diversity) as measured by Observed species and Shannon Diversity indices was lower in samples from mongooses trapped in the more densely inhabited and commercialized area (Figure 6) and significantly different from samples from mongooses trapped at the quarry (GR_PB vs. GR_QA, *p* = 0.008), the protected open wood- and shrubland (GR_PB vs. GR_PH, *p* = 0.001), and a coastal and mixed shrubland area (GR_PB vs. GR_ST, *p* = 0.020).

Samples from mongooses trapped on the peninsular part of the island separated from the other sampling locations in the UniFrac unweighted beta diversity analysis (*p* < 0.05) but not in the weighted analysis (Figure S2). This suggests that the largest variation in samples from the peninsula was associated with specific taxa occurring at a low relative abundance. Consequently, no distinct clustering of samples per trapping location could be observed when taking into account both richness and evenness of the bacterial communities.

**Figure 7:**
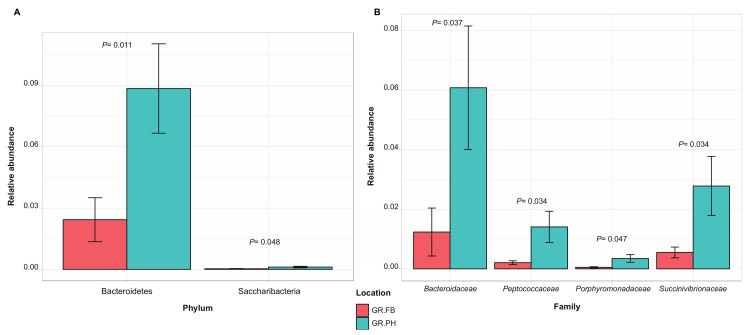
Discriminating taxa significantly different in relative abundance (average ± SEM) at phylum (**A**) and family (**B**) level between fecal samples from small Indian mongooses trapped in a protected open wood- and scrubland (GR.PH, blue) and trapped in a dense inhabited and commercialized area (GR.FB, red) (*p* < 0.05, between-group *t*-test analysis).

Across the different trapping locations, samples shared 43% of OTUs. Comparison of the relative abundance of taxa between trapping locations revealed a significant variation between samples from mongooses trapped at the protected open wood- and scrubland and those trapped at the dense inhabited and commercialized area. At the phylum level, Bacteroidetes were enriched in the fecal microbial community from samples retrieved in the protected wood- and scrubland, and at the family level, four discriminating families have been identified, i.e., *Bacteroidaceae*, *Succinivibrionaceae*, *Peptococcaceae*, and *Porphyromonoadaceae* (Figure 7).

### 3.4. Functional Characterization of Bacterial Communities in Small Indian Mongooses

The greatest number of predicted genes among level 1 KEGG Orthology (KO) categories that were assigned a function encoded proteins involved in “metabolism” (55.69%). Among second-level functional categories, the highest functional abundance (>10% of gene counts) was observed for “amino acid metabolism” (11.27%), “carbohydrate metabolism” (13.20%), and “membrane transport” (17.51%) (Table S5).

Several amino acid synthesis pathways were predicted, of which the most prominent inferred functional assignments were (expressed as % of total predicted genes for amino acid metabolism) the arginine biosynthesis (12.79%), the glycine, serine, and threonine metabolism (8.60%), the cysteine and methionine metabolism (9.69%), and the valine, leucine, and isoleucine synthesis (7.61%) pathways. The most abundant pathways predicted for carbohydrate metabolism (expressed as % of total predicted genes for carbohydrate metabolism) were the citrate cycle (5.26%), the galactose metabolism (6.45%), the ascorbate and aldarate metabolism (1.54%), the starch and sucrose metabolism (8.78%), the pyruvate metabolism (10%), and the propanoate metabolism (5.77%) pathways.

We have tested the correlation between the most abundant phyla and families and the level 1 functional categories. The abundances of amino acid metabolism were positively correlated with the 10 most abundant phyla (r = 0.70, *p* < 0.05) but negatively correlated with the 10 most abundant families (r = −0.74, *p* < 0.05). The abundances of carbohydrate metabolism were also positively correlated with the 10 most abundant phyla (r = 0.69, *p* < 0.05) but there was no significant association with the 10 most abundant families (r = 0.13, *p* = 0.733).

The phyla Firmicutes and Proteobacteria were the two most contributing taxa towards amino metabolic pathways. *Bacillaceae* contributed to all predicted genes for the cysteine and methionine metabolism whereas *Rhodobiaceae* and *Sphingomonadaceae*, both belonging to the Proteobacteria, contributed most to the functional predictions of the cysteine and methionine metabolism. *Lachnospiraceae* and *Clostridiaceae* were the highest contributors to the predictive genes for valine, leucine, and isoleucine biosynthesis and *Peptostreptococcaceae* to those of the glycine, serine, and threonine metabolism pathways. The phyla Firmicutes and Fusobacteria were the two most contributing taxa towards carbohydrate metabolic pathways. *Fusobacteriaceae* contributed to the majority of predicted genes for the citrate cycle. *Lachnospiraceae*, *Clostridiaceae*, and *Fusobacteriaceae* contributed the most to galactose metabolism, pyruvate metabolism, propanoate metabolism, and the ascorbate and aldarate metabolism pathways. Finally, *Cellulomonadaceae* contributed the majority of the predictive genes for the starch and sucrose metabolism which is only marginally represented in the functional inference analysis.

**Figure 8:**
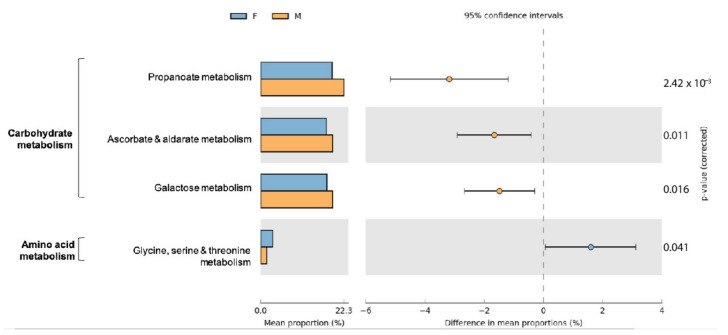
Extended error bar plot indicating the functional differences of gut microbiota in male and female small Indian mongooses. KEGG categories were obtained from 16S rRNA gene sequences using PICRUSt. Only significant differences between two groups for pathways contributing to amino acid metabolism and carbohydrate metabolism are depicted (Welch’s *t*-test, *p* < 0.05).

Differences in functional community profiles were further examined between the different sampling sites and both sexes. Within sampling sites and sexes, little variation is seen in the abundances and distributions of second-level KO functional gene annotations (Figure S3). The proportion of sequences attributed to carbohydrate metabolism and amino acid metabolism differed only marginally between males and females (*p* < 0.05) (Figure S4). Pathways contributing to carbohydrate metabolism and amino acid metabolism with a significant difference between male and female are summarized in Figure 8. We detected predicted increases in genes related to glycine, serine, and threonine metabolism in females and overall a predicted increase in three pathways contributing to carbohydrate metabolism in males. No significant differences between functional predictions for amino acid or carbohydrate metabolism were detected between the nine sampling sites.

## 4. Discussion

In this study, we generated the first extensive sequence library highlighting the taxonomic diversity and functional potential of the bacterial gut microbiota of small Indian mongooses (*Urva auropunctata*), an invasive species free-roaming on Caribbean islands. We reinforce the notion that the gut bacterial composition is reflective of both feeding ecology and host evolutionary history [31,32]. Small Indian mongooses (Carnivora, *Herpestidae*) are opportunistic terrestrial carnivores and their gut microbial diversity echoes a carnivore-like signature [33] with a dominant abundance of Firmicutes (54.96%), followed by Proteobacteria (13.98%) and Fusobacteria (12.39%), and a relatively minor contribution of Actinobacteria (10.4%) and Bacteroidetes (6.40%). Within the Firmicutes, proteolytic bacteria such as *Blautia* (*Lachnospiraceae*), *Peptoclostridium* (*Peptostreptococcaceae*), and *Clostridium* (*Clostridiaceae*) were central. In particular, *Clostridium* spp. have been associated in carnivores with the butyrate kinase butyrate-synthesis pathway, which allows the production of butyrate from protein [34]. This corroborates recent co-occurrence analyses across 128 different vertebrates underpinning that *Clostridiaceae*, *Lachnospiraceae*, and *Enterobacteriaceae* (Proteobacteria) are more prevalent in carnivores compared to herbivores and omnivores [31].

We also calculated a Proteobacteria to Bacteroidetes ratio of 2.2, which is comparable to the ratio of 3 reported in Egyptian mongooses [12]. However, the Firmicutes to Bacteroidetes ratio of 8.59 is lower compared to the ratio obtained for other carnivores such as the Tasmanian devil [35], spotted hyena [36], cheetah [37], and the Egyptian mongoose [12].

Interestingly, in comparison to Egyptian mongooses, phyla Proteobacteria and Fusobacteria contributed each up to 10% more to the fecal bacterial composition in small Indian mongooses, thereby relatively decreasing the dominance of Firmicutes. Despite technical artifacts inherent to differing experimental designs between both studies, we speculate that dietary differences across Egyptian and small Indian mongooses drive the observed dissimilarities in their gut microbial composition. A recent large-scale study in Portugal has uncovered the Egyptian mongoose’s diet which is, in terms of consumed biomass, mainly composed of mammals (63.06%), reptiles (16.27%), amphibians (7.86%), invertebrates (6.62%), and carrion (4.09%), with a minor inclusion of fish (1.00%), plant material (0.66%), eggs (0.34%), and birds (0.09%) [14]. 

A comprehensive study on the feeding patterns of mongooses inhabiting the Caribbean islands is still lacking, but an initial exploration of the diet of mongooses on the island St John (United States Virgin Islands) suggested that their diets consist of approximately 19–30% arthropods and 59–69% vertebrates, with a heavy predation on eggs of the brown pelican, the green sea turtle, and multiple species of quail dove, next to herpetofauna (i.e., anoles, geckos), crustaceans, fruits, and plants [11]. Therefore, the small Indian mongooses from our study rely more heavily on eggs, marine-derived nutrients, fruits, and smaller non-mammalian prey items compared to Egyptian mongooses. These varied protein, fat, animal, and vegetable fiber sources likely shape the composition of the Caribbean small Indian mongoose gut microbiota, increasing the relative contribution of Proteobacteria and Fusobacteria. The latter have been known to specifically in carnivores produce butyrate from protein sources and increase with increased dietary protein content [38]. 

The relative abundance of *Fusobacterium* spp. was relatively higher in samples collected from mongooses trapped at coastal trapping sites. This could not only result from a higher protein intake (eggs from sea turtles and coastal birds, fish, crustaceans) but also from inoculation of Fusobacteria associated with marine prey items that hold large numbers of Fusobacteria in their gastrointestinal tract [39]. Additionally, samples collected from mongooses inhabiting dense scrub- and woodland areas were significantly enriched in members of the phylum Bacteroidetes which is reflective of diets rich in vegetable and animal fiber [40] compared to samples from mongooses trapped in dense inhabited and commercialized areas where they are scavenging garbage and food left-overs. Although there was no significant difference in the predicted metabolic pathways between sampling locations, data suggest that subtle differences in bacterial composition at different taxonomic levels could be driven by fine-scale variations in feeding patterns. Because of the mongooses’ dietary plasticity on Caribbean islands, they may affect several trophic levels by feeding on both vertebrates, invertebrates, and plants. It has been suggested that mongooses likely have a preference for prey found close to the site of capture and that their larger movements are more exploratory in nature [11]. To this end, radio-tracking studies can enable further insights into the mongoose’s migratory and feeding patterns and their potential impact on trophic dynamics in the region.

Sex differences in gut microbial composition have been reported in many different species [41]. When comparing gut microbial communities across sexes, the proportional abundance of Gammaproteobacteria, more specifically *Enterobacteriaceae*, was significantly higher in males compared to females. Whereas an enrichment in *Enterobacteriaceae* reflects a carnivore-like microbiota profile, the reasons for the compositional difference between sexes is yet unclear. Beta diversity analyses, however, showed no significant difference across sexes, suggesting similar taxa make up their core microbial composition with variable relative proportions of taxa. The differential functional profile between sexes did not align with previous findings in Egyptian mongooses, whereby male Egyptian mongooses showed a higher abundance of catabolic pathways of valine, leucine, and isoleucine amino acids and females exhibited a higher abundance of galactose metabolic pathways. In fact, the galactose metabolism was slightly more abundant in the male small Indian mongoose hosts though the *Bacteroides* spp., which normally perform this pathway, were not overrepresented. Though represented in high gene abundance, the valine, leucine, and isoleucine biosynthesis was not significantly different between sexes. Neither were the citrate cycle modules to which mostly the *Fusobacteria* contributed in both sexes. Another abundant pathway as gleaned from the 16S rRNA dataset was the propanoate/propionate metabolism. This functional trait potentially reflects the carnivorous nature of mongooses, as propionate absorbed from the colon can act as a gluconeogenic substrate in carnivores [42]. However, in contrast with findings in Egyptian mongooses [12], no sex-specific strategies in energy production were further observed in small Indian mongooses.

Microbial symbiosis is a foundational principle for the competitive success of invasive species and their health, productivity, and adaptive capacity have been linked in various ways to their microbiomes [43]. Whereas this study provides the first insights into the fecal microbial profile of one of the world’s worst invasive species, the small Indian mongoose, we should consider to further integrate such data in invasive species management, disturbance ecology and invasion biology. Traditionally, mongooses have been studied in their role as vector of pathogens of human and veterinary importance, with an emphasis on the transmission of zoonotic pathogens [7,44]. However, few have considered the effect of invasive microorganisms carried by the introduced species on native species [45] or on the microbial components and processes of ecosystems. These studies are challenging as the effects of such influences are secondary and hard to determine. Improving our knowledge on the bioecology of invasive species, including their associated microbiome, will therefore further aid in fully comprehend their ecology in light of behavior, diet, geographic features, and their invasive success and potential expansion.

## Data Availability

The data presented in this study are openly available in BioProject accession number PRJNA688145 and in Supplementary File 1.

## Supplementary Materials

The following are available online at https://www.mdpi.com/2076-2607/9/3/465/s1, Figure S1: Principal component analysis (PCoA) of the gut microbiota of male (red, *n* = 28) and female (green, *n* = 32) small Indian mongooses, based on unweighted UniFrac distances. Figure S2: (A) Principal component analysis (PCoA) of the gut microbiota of fecal samples from 60 small Indian mongooses trapped at nine different locations, based on unweighted and weighted UniFrac distances, (B) Boxplots of unweighted and weighted UniFrac distances, with significant difference in unweighted UniFrac between samples collected at the peninsula and five other locations. Figure S3: Percentage of level 2 KEGG Orthology (KO) functions among PICRUSt functional predictions per sampling site (A) and sex (B). Figure S4: Extended error bar plots show significant differences between mean proportions of functional predictions at KEGG level 2 “Amino acid metabolism” and “Carbohydrate metabolism”. Table S1: Metadata of free-roaming wild populations of small Indian mongooses (*Urva auropunctata*) trapped on the Caribbean island St Kitts, stratified by trapping location. Table S2: Study trapping locations for free-roaming wild populations of small Indian mongooses (*Urva auropunctata*) on the Caribbean island St Kitts as defined by land cover and forest formation. Table S3: Data on clean reads after quality filtering and chimera removal. Table S4: Alpha diversity indices for 60 fecal samples from 60 small Indian mongooses, resulting from V3–V4 16S rRNA amplicon gene sequencing, with sorting of high-quality reads into operational taxonomic units at 97% sequence identity cutoff. Table S5: Percentages of predicted gene counts assigned to second level KO categories in the metagenomic dataset retrieved from PICRUSt predictions on 16S rRNA gene dataset from 60 fecal samples from small Indian mongooses. Supplementary File 1: Fasta file.

## References

[1] Trevelline B.K., Fontaine S.S., Hartup B.K., Kohl K.D. (2019). Conservation biology needs a microbial renaissance: A call for the consideration of host-associated microbiota in wildlife management practices. Proc. R. Soc. B.

[2] West A.G., Waite D.W., Deines P., Bourne D.G., Digby A., Mckenzie V.J., Taylor M.W. (2019). The microbiome in threatened species conservation. Biol. Conserv..

[3] (2020). Global Invasive Species Database. http://www.iucngisd.org/gisd/100_worst.php.

[4] Veron G., Patou M.L., Pothet G., Simberloff D., Jennings A.P. (2007). Systematic status and biogeography of the Javan and small Indian mongooses (Herpestidae, Carnivora). Zool. Scr..

[5] Horst G.R., Hoagland D.B., Kilpatrick C.W. (2001). The mongoose in the West Indies: The biogeography and population biology of an introduced species. Biogeography of the West Indies: Patterns and Perspectives.

[6] Hoagland D., Horst G., Kilpatrick C., Wood C. (1989). Biogeography and population biology of the mongoose in the West Indies. Biogeography of the West Indies: Past, Present and Future.

[7] Cheng T., Halper B., Siebert J., Cruz-Martinez L., Chapwanya A., Kelly P., Ketzis J.K., Vessell J., Köster L., Yao C. (2018). Parasites of small Indian mongoose, Herpestes auropunctatus, on St. Kitts, West Indies. Parasitol. Res..

[8] Berentsen A.R., Pitt W.C., Sugihara R.T. (2017). Ecology of the small indian mongoose (herpestes auropunctatus) in North America. Ecology and Management of Terrestrial Vertebrate Invasive Species in the United States.

[9] Louppe V., Leroy B., Herrel A., Veron G. (2020). The globally invasive small Indian mongoose Urva auropunctata is likely to spread with climate change. Sci. Rep..

[10] Shekhar K. (2003). The status of mongooses in central India. Small Carniv. Conserv..

[11] Pieter A.P., Powers K.E., Hyzy B.A. (2016). Initial explorations into the feeding ecology of the invasive small Indian mongoose in the Caribbean using stable isotope analyses. Bios.

[12] Pereira A.C., Bandeira V., Fonseca C., Cunha M.V. (2020). Egyptian mongoose (Herpestes ichneumon) gut microbiota: Taxonomical and functional differences across sex and age classes. Microorganisms.

[13] Patou M.L., Mclenachan P.A., Morley C.G., Couloux A., Jennings A.P., Veron G. (2009). Molecular phylogeny of the Herpestidae (Mammalia, Carnivora) with a special emphasis on the Asian Herpestes. Mol. Phylogenet. Evol..

[14] Bandeira V., Virgos E., Carvalho J., Barros T., Cunha M.V., Fonseca C. (2018). Diet footprint of Egyptian mongoose along ecological gradients: Effects of primary productivity and life history traits. Mamm. Biol..

[15] Moeller A.H., Suzuki T.A., Lin D., Lacey E.A., Wasser S.K., Nachman M.W. (2017). Dispersal limitation promotes the diversification of the mammalian gut microbiota. Proc. Natl. Acad. Sci. USA.

[16] Ley R.E., Hamady M., Lozupone C., Turnbaugh P.J., Ramey R.R., Bircher J.S., Schlegel M.L., Tucker T.A., Schrenzel M.D., Knight R. (2008). Evolution of mammals and their gut microbes. Science.

[17] Helmer E.H., Kennaway T.A., Pedreros D., Clark M. (2008). Distributions of land cover and forest formations for St. Kitts, Nevis, St. Eustatius, Grenada and Barbados from satellite imagery. Caribb. J. Sci..

[18] Kumar J., Kumar M., Gupta S., Ahmed V., Bhambi M., Pandey R., Chauhan N.S. (2016). An Improved Methodology to Overcome Key Issues in Human Fecal Metagenomic DNA Extraction. Genom. Proteom. Bioinform..

[19] Martin M. (2011). Cutadapt removes adapter sequences from high-throughput sequencing reads. EMBnet J..

[20] Bokulich N.A., Subramanian S., Faith J.J., Gevers D., Gordon J.J., Knight R., Mills D.A., Caporaso J.G. (2013). Quality-filtering vastly improves diversity estimates from Illumina amplicon sequencing. Nat. Methods.

[21] Edgar R.C., Haas B.J., Clemente J.C., Quince C., Knight R. (2011). UCHIME improves sensitivity and speed of chimera detection. Bioinformatics.

[22] Edgar R. (2013). UPARSE: Highly accurate OTU sequences from microbial amplicon reads. Nat. Methods.

[23] Quast C., Pruesse E., Yilmaz P., Gerken J., Schweer T., Yarza P., Peplies J., Glöckner F.O. (2013). The SILVA ribosomal RNA gene database project: Improved data processing and web-based tools. Nucleic Acids Res..

[24] Edgar R.C. (2004). MUSCLE: Multiple sequence alignment with high accuracy and high throughput. Nucleic Acids Res..

[25] Caporaso J.G., Kuczynski J., Stombaugh J., Bittinger K., Bushman F.D., Costello E.K., Fierer N., Peña A.G., Goodrich J.K., Gordon J.I. (2010). QIIME allows analysis of high- throughput community sequencing data Intensity normalization improves color calling in SOLiD sequencing. Nat. Methods.

[26] R Core Team R: A Language and Environment for Statistical Computing. http://www.r-project.org.

[27] Good I.J. (1953). The population frequencies of species and the estimation of population parameters. Biometrika.

[28] Langfelder P., Horvath S. (2012). Fast R Functions for Robust Correlations and Hierarchical Clustering. J. Stat. Softw..

[29] Wickham H. (2016). ggplot2: Elegant Graphics for Data Analysis.

[30] Langille M.G.I., Zaneveld J., Caporaso J.G., McDonald D., Knights D., Reyes J.A., Clemente J.C., Burkepile D.E., Vega Thurber R.L., Knight R. (2013). Predictive functional profiling of microbial communities using 16S rRNA marker gene sequences. Nat. Biotechnol..

[31] Youngblut N.D., Reischer G.H., Walters W., Schuster N., Walzer C., Stalder G., Ley R.E., Farnleitner A.H. (2019). Host diet and evolutionary history explain different aspects of gut microbiome diversity among vertebrate clades. Nat. Commun..

[32] Muegge B.D., Kuczynski J., Knights D., Clemente J.C., González A., Fontana L., Henrissat B., Knight R., Gordon J.I. (2011). Diet drives convergence in gut microbiome functions across mammalian phylogeny and within humans. Science.

[33] Nishida A.H., Ochman H. (2018). Rates of Gut Microbiome Divergence in Mammals. Mol. Ecol..

[34] Vital M., Gao J., Rizzo M., Harrison T., Tiedje J.M. (2015). Diet is a major factor governing the fecal butyrate-producing community structure across Mammalia, Aves and Reptilia. ISME J..

[35] Cheng Y., Fox S., Pemberton D., Hogg C., Papenfuss A.T., Belov K. (2015). The Tasmanian devil microbiome-implications for conservation and management. Microbiome.

[36] Chen L., Liu M., Zhu J., Gao Y., Sha W., Ding H., Jiang W., Shenping W. (2020). Age, gender, and feeding environment influence fecal microbial diversity in Spotted Hyenas (Crocuta crocuta). Curr. Microbiol..

[37] Becker A.A.M.J., Hesta M., Hollants J., Janssens G.P.J., Huys G. (2014). Phylogenetic analysis of faecal microbiota from captive cheetahs reveals underrepresentation of Bacteroidetes and Bifidobacteriaceae. BMC Microbiol..

[38] Pilla R., Suchodolski J.S. (2020). The Role of the Canine Gut Microbiome and Metabolome in Health and Gastrointestinal Disease. Front. Vet. Sci..

[39] Ghanbari M., Kneifel W., Doming K.J. (2015). A new view of the fish gut microbiome: Advances from next-generation sequencing. Aquaculture.

[40] Ferrario C., Statello R., Carnevali L., Mancabelli L., Milani C., Mangifesta M., Duranti S., Lugli G.A., Jimenez B., Lodge S. (2017). How to Feed the Mammalian Gut Microbiota: Bacterial and Metabolic Modulation by Dietary Fibers. Front. Microbiol..

[41] Kim Y.S., Unno T., Kim B.Y., Park M.S. (2020). Sex differences in gut microbiota. World J. Mens. Health.

[42] Verbrugghe A., Hesta M., Daminet S., Polis I., Holst J.J., Buyse J., Wuyts B., Janssens G.P.J. (2012). Propionate absorbed from the colon acts as gluconeogenic substrate in a strict carnivore, the domestic cat (Felis catus). J. Anim. Physiol. Anim. Nutr..

[43] Kowalski K.P., Bacon C., Bickford W., Braun H., Clay K., Leduc-Lapierre M., Lillard E., McCormick M.K., Nelson E., Torres M. (2015). Advancing the science of microbial symbiosis to support invasive species management: A case study on Phragmites in the Great Lakes. Front. Microbiol..

[44] Shiokawa K., Llanes A., Hindoyan A., Cruz-Martinez L., Welcome S., Rajeev S. (2019). Peridomestic small Indian mongoose: An invasive species posing as potential zoonotic risk for leptospirosis in the Caribbean. Acta Trop..

[45] Bahrndorff S., Alemu T., Alemneh T., Lund Nielsen J. (2016). The Microbiome of Animals: Implications for Conservation Biology. Int. J. Genom..

