# Supplementary material for: Unraveling the Gut Microbiome of the Invasive Small Indian Mongoose (Urva auropunctata) in the Caribbean
Source: Microorganisms. 2021 Feb 24;9(3):465. doi: 10.3390/microorganisms9030465 (PMC7996244; doi:10.3390/microorganisms9030465)
Supplement: Supplementary file 1 [file microorganisms-09-00465-s001.zip › Proof_Supplementary Materials_ABecker/Supplementary_Table5_v1.docx]

**Supplementary Table 5.** Percentages of predicted gene counts assigned to second level KO categories in the metagenomic dataset retrieved from PICRUSt predictions on 16S rRNA gene dataset from 60 fecal samples from small Indian mongooses.

| Function^1^ | Total dataset (n=60) |
| --- | --- |
| Metabolism^2^ | **55.69** |
| Amino acid metabolism | 11.27 |
| Biosynthesis of other secondary metabolites | 1.03 |
| Carbohydrate metabolism | 13.20 |
| Energy metabolism | 6.61 |
| Enzyme families | 2.52 |
| Glycan Biosynthesis and metabolism | 2.05 |
| Lipid metabolism | 3.44 |
| Metabolism of cofactors and vitamins | 4.93 |
| Metabolism of other amino acids | 1.82 |
| Metabolism of terpenoids and polyketides | 1.92 |
| Nucleotide metabolism | 4.34 |
| Xenobiotics biodegradation and metabolism | 2.56 |
| Genetic Information Processing^2^ | **21.33** |
| Folding, sorting and degradation | 2.62 |
| Replication and repair | 9.30 |
| Transcription | 3.61 |
| Translation | 5.80 |
| Environmental Information Processing^2^ | **19.82** |
| Membrane transport | 17.51 |
| Signal transduction | 2.12 |
| Signaling molecules and interaction | 0.20 |
| Cellular Processes^2^ | **3.15** |
| Cell growth and death | 0.53 |
| Cell motility | 2.39 |
| Transport and catabolism | 0.23 |

^1^Functional categories for which no reads were assigned are omitted.

^2^Predicted function assigned at level 1 KO category.
