# Supplementary material for: Unraveling the Gut Microbiome of the Invasive Small Indian Mongoose (Urva auropunctata) in the Caribbean
Source: Microorganisms. 2021 Feb 24;9(3):465. doi: 10.3390/microorganisms9030465 (PMC7996244; doi:10.3390/microorganisms9030465)
Supplement: Supplementary file 1 [file microorganisms-09-00465-s001.zip › Proof_Supplementary Materials_ABecker/Supplementary_Table3_proof.docx]

**Supplementary Table 3.** Data on clean reads after quality filtering and chimera removal.

| **Animal code** | **Raw reads** | **Clean reads** | **Base (nt)** | **AvgLength**  **(nt)** | **Q20** | **GC%** | **Effective %** |
| --- | --- | --- | --- | --- | --- | --- | --- |

| M20 | 266997 | 160129 | 66519959 | 415 | 88.04 | 52.12 | 59.97 |
| --- | --- | --- | --- | --- | --- | --- | --- |
| M21 | 180724 | 160182 | 68267334 | 426 | 83.82 | 55.64 | 88.63 |
| M22 | 186058 | 160295 | 66623636 | 415 | 81.63 | 54.07 | 86.15 |
| M23 | 200686 | 160168 | 67389598 | 420 | 87.9 | 53.62 | 79.81 |
| M24 | 182762 | 160171 | 66115972 | 412 | 86.94 | 54 | 87.64 |
| M25 | 184980 | 160065 | 65459205 | 408 | 86.27 | 53.74 | 86.53 |
| M26 | 204982 | 160126 | 65532977 | 409 | 86 | 54.56 | 78.12 |
| M27 | 169344 | 154553 | 63599392 | 411 | 86.69 | 53.97 | 91.27 |
| M28 | 239298 | 160068 | 66274445 | 414 | 87.23 | 50.74 | 66.89 |
| M29 | 188567 | 160348 | 65712969 | 409 | 86.29 | 54.52 | 85.04 |
| M30 | 206223 | 160197 | 65407710 | 408 | 86.05 | 54.22 | 77.68 |
| M31 | 143237 | 115265 | 47842395 | 415 | 86.82 | 56.96 | 80.47 |
| M32 | 191755 | 160199 | 66474287 | 414 | 87.59 | 53.16 | 83.54 |
| M33 | 193761 | 160068 | 66767126 | 417 | 82.19 | 53.53 | 82.61 |
| M35 | 196150 | 160133 | 66464564 | 415 | 87.48 | 53.35 | 81.64 |
| M37 | 181659 | 160056 | 65226606 | 407 | 88.34 | 52.21 | 88.11 |
| M38 | 163464 | 149676 | 61846532 | 413 | 81.74 | 52.65 | 91.57 |
| M39 | 175465 | 160101 | 65920061 | 411 | 84.51 | 50.22 | 91.24 |
| M40 | 147627 | 129064 | 53497058 | 414 | 85.42 | 55.22 | 87.43 |
| M41 | 185434 | 169634 | 69925461 | 412 | 82.44 | 53.41 | 91.48 |
| M42 | 132650 | 113168 | 46553008 | 411 | 81.72 | 55.44 | 85.31 |
| M43 | 190212 | 171432 | 70831727 | 413 | 85.31 | 52.08 | 90.13 |
| M44 | 175383 | 160274 | 66378621 | 414 | 82.91 | 51.32 | 91.39 |
| M45 | 157325 | 142382 | 58878769 | 413 | 82 | 52.97 | 90.5 |
| M46 | 108546 | 93067 | 39019563 | 419 | 82.52 | 55.55 | 85.74 |
| M47 | 114275 | 93349 | 38860953 | 416 | 83.68 | 55.65 | 81.69 |
| M48 | 174454 | 160031 | 66924567 | 418 | 82.45 | 52.64 | 91.73 |
| M49 | 124478 | 105256 | 43886467 | 416 | 81.76 | 56.13 | 84.56 |
| M50 | 183220 | 160247 | 68012990 | 424 | 84.02 | 54.75 | 87.46 |
| M51 | 112112 | 97925 | 40702453 | 415 | 81.25 | 55.61 | 87.35 |
| M52 | 180485 | 160117 | 65334869 | 408 | 85.54 | 53.87 | 88.71 |
| M53 | 186383 | 160187 | 65364873 | 408 | 82.13 | 55.38 | 85.95 |
| M55 | 186422 | 160061 | 66009310 | 412 | 83.06 | 52.57 | 85.86 |
| M56 | 178967 | 160098 | 65751358 | 410 | 84.25 | 53.61 | 89.46 |
| M57 | 173956 | 160062 | 65984901 | 412 | 88.48 | 52.07 | 92.01 |
| M58 | 174787 | 160189 | 66114907 | 412 | 86.81 | 52.46 | 91.65 |
| M59 | 166826 | 144909 | 59747984 | 412 | 85.94 | 54.1 | 86.86 |
| M60 | 177071 | 160156 | 66272604 | 413 | 87.89 | 53.29 | 90.45 |
| M61 | 169550 | 160309 | 66493100 | 414 | 88.57 | 52.49 | 94.55 |
| M62 | 172057 | 160150 | 65465983 | 408 | 90.91 | 49.68 | 93.08 |
| M63 | 179859 | 160315 | 65912732 | 411 | 87.79 | 52.95 | 89.13 |
| M64 | 105445 | 89041 | 37011226 | 415 | 86.06 | 56.74 | 84.44 |
| M65 | 168050 | 160316 | 65932018 | 411 | 86.43 | 53.01 | 95.4 |
| M66 | 183647 | 160318 | 65802841 | 410 | 87.55 | 52.82 | 87.3 |
| M67 | 125470 | 102625 | 42602812 | 415 | 88.21 | 56.9 | 81.79 |
| M68 | 170126 | 154763 | 63598264 | 410 | 86.04 | 54.4 | 90.97 |
| M69 | 151996 | 134197 | 55707223 | 415 | 86.29 | 56.01 | 88.29 |
| M70 | 130671 | 117742 | 48951279 | 415 | 82.48 | 51.41 | 90.11 |
| M71 | 102565 | 88421 | 36850404 | 416 | 84.08 | 56.76 | 86.21 |
| M72 | 203892 | 160156 | 66005010 | 412 | 86.96 | 55.63 | 78.55 |
| M73 | 181951 | 160033 | 66220145 | 413 | 88.11 | 52.13 | 87.95 |
| M74 | 173616 | 160217 | 65671375 | 409 | 81.13 | 52.47 | 92.28 |
| M75 | 159008 | 149774 | 61965153 | 413 | 83.05 | 52.08 | 94.19 |
| M76 | 129601 | 122753 | 50683276 | 412 | 81.47 | 52.41 | 94.72 |
| M77 | 166202 | 153353 | 63458085 | 413 | 84.58 | 50.83 | 92.27 |
| M78 | 179764 | 160171 | 66601332 | 415 | 87.45 | 53.68 | 89.1 |
| M80 | 184256 | 160168 | 65714324 | 410 | 86.07 | 54.09 | 86.93 |
| M81 | 118374 | 96321 | 40023736 | 415 | 80.54 | 51.92 | 81.37 |
| M82 | 204904 | 160170 | 65774830 | 410 | 85.27 | 53.58 | 78.17 |
| M83 | 104435 | 85988 | 35791169 | 416 | 83.43 | 56.61 | 82.34 |

*Raw reads after filtration; clean reads after chimera removal; Q20 = percentage of base quantity that is greater than 20; Effective(%)= percentage of clean reads raw reads*
