# Supplementary material for: Unraveling the Gut Microbiome of the Invasive Small Indian Mongoose (Urva auropunctata) in the Caribbean
Source: Microorganisms. 2021 Feb 24;9(3):465. doi: 10.3390/microorganisms9030465 (PMC7996244; doi:10.3390/microorganisms9030465)
Supplement: Supplementary file 1 [file microorganisms-09-00465-s001.zip › Proof_Supplementary Materials_ABecker/Supplementary_Table4_v1.docx]

**Supplementary Table 4.** Alpha diversity indices for 60 fecal samples from 60 small Indian mongooses, resulting from V3-V4 16S rRNA amplicon gene sequencing, with sorting of high-quality reads into operational taxonomic units at 97% sequence identity cutoff.

| **Animal code** | **Observed Species** | **Chao** | **ACE index** | **Shannon Diversity Index (H’)** |
| --- | --- | --- | --- | --- |
| M20 | 404 | 589.389 | 613.49 | 4.433 |
| M21 | 381 | 566.119 | 604.639 | 4.291 |
| M22 | 768 | 964.941 | 991.358 | 5.717 |
| M23 | 1701 | 2192.181 | 2239.508 | 6.042 |
| M24 | 1016 | 1276.65 | 1280.403 | 5.459 |
| M25 | 885 | 1197.248 | 1207.46 | 5.017 |
| M26 | 659 | 907.11 | 958.315 | 6.182 |
| M27 | 899 | 1196.007 | 1239.932 | 5.968 |
| M28 | 565 | 664.917 | 663.748 | 6.489 |
| M29 | 1400 | 1623.779 | 1658.618 | 5.111 |
| M30 | 631 | 992.5 | 993.695 | 5.796 |
| M31 | 2138 | 2510.223 | 2470.502 | 8.926 |
| M32 | 704 | 1043.082 | 973.91 | 5.657 |
| M33 | 809 | 1079.153 | 1107.799 | 6.493 |
| M35 | 1723 | 2148.04 | 2154.538 | 6.451 |
| M37 | 494 | 682.263 | 676.048 | 5.168 |
| M38 | 987 | 1174.633 | 1204.5 | 6.351 |
| M39 | 928 | 1299.106 | 1309.809 | 5.587 |
| M40 | 2148 | 2504.68 | 2534.174 | 8.281 |
| M41 | 915 | 1144.185 | 1196.493 | 5.674 |
| M42 | 1391 | 1770.822 | 1737.074 | 5.924 |
| M43 | 1677 | 229.768 | 2184.683 | 6.528 |
| M44 | 705 | 851.879 | 867.396 | 6.569 |
| M45 | 855 | 1024.759 | 1049.104 | 6.633 |
| M46 | 1554 | 1679.525 | 1698.972 | 6.921 |
| M47 | 1874 | 1972.312 | 1979.578 | 8.608 |
| M48 | 925 | 1186.074 | 1217.373 | 6.664 |
| M49 | 2098 | 2252.124 | 2271.011 | 9.04 |
| M50 | 549 | 747.446 | 773.464 | 5.187 |
| M51 | 2063 | 2231.644 | 2241.409 | 8.658 |
| M52 | 826 | 1100.103 | 1178.915 | 3.364 |
| M53 | 518 | 671.381 | 734.344 | 4.295 |
| M55 | 614 | 761.439 | 799.398 | 5.906 |
| M56 | 442 | 587 | 623.212 | 4.069 |
| M57 | 670 | 968.5 | 941.78 | 5.943 |
| M58 | 758 | 1066.111 | 1060.117 | 6.535 |
| M59 | 1374 | 1645.736 | 1717.691 | 6.225 |
| M60 | 1184 | 1644.159 | 1625.663 | 6.439 |
| M61 | 920 | 1068.688 | 1048.885 | 6.328 |
| M62 | 448 | 564.644 | 592.783 | 3.265 |
| M63 | 730 | 937.03 | 981.305 | 6.617 |
| M64 | 2021 | 2179.814 | 2206.17 | 8.94 |
| M65 | 702 | 895.147 | 918.867 | 5.512 |
| M66 | 659 | 939.16 | 968.708 | 5.336 |
| M67 | 2007 | 2148.479 | 2195.151 | 8.834 |
| M68 | 1463 | 1972.06 | 2038.582 | 5.978 |
| M69 | 2305 | 2746.752 | 2744.351 | 8.867 |
| M70 | 881 | 1171.049 | 1240.773 | 5.609 |
| M71 | 1934 | 2046.149 | 2073.503 | 8.918 |
| M72 | 1951 | 2196.464 | 2247.672 | 7.754 |
| M73 | 946 | 1090.008 | 1070.413 | 6.064 |
| M74 | 518 | 687.382 | 669.065 | 4.691 |
| M75 | 622 | 771 | 782.078 | 6.084 |
| M76 | 705 | 886.116 | 893.975 | 6.304 |
| M77 | 875 | 1194.446 | 1188.225 | 6.426 |
| M78 | 829 | 1122.834 | 1197.647 | 5.008 |
| M80 | 994 | 1356.312 | 1399.857 | 5.318 |
| M81 | 795 | 884.554 | 898.052 | 7.276 |
| M82 | 1134 | 1526.08 | 1541.797 | 5.883 |
| M83 | 2047 | 2080.225 | 2132.525 | 9.108 |
