# Supplementary material for: Unraveling the Gut Microbiome of the Invasive Small Indian Mongoose (Urva auropunctata) in the Caribbean
Source: Microorganisms. 2021 Feb 24;9(3):465. doi: 10.3390/microorganisms9030465 (PMC7996244; doi:10.3390/microorganisms9030465)
Supplement: Supplementary file 1 [file microorganisms-09-00465-s001.zip › Proof_Supplementary Materials_ABecker/Supplementary_Table1_proof.docx]

**Supplementary Table 1.** Metadata of free-roaming wild populations of small Indian mongooses (*Urva auropunctata*) trapped on the Caribbean island St Kitts, stratified by trapping location. Upon anesthesia and euthanasia, routine necropsies were performed. Body condition score (BCS) was measured according to a 5-scale scoring chart (1: emaciated, 2: underconditioned, 3: well-conditioned, 4: overconditioned, 5: obese) evaluating the pronunciation and segmentation of the vertebral column, rib cage, dorsal pelvis, sit bones, tail and overall fat cover. Animals were placed into four age classes (1: juvenile (<1 year), 2: young adult, 3: adult, 4: senior) by relying on tooth wearing, skull size and color of the eyes.

| **Animal code** | **Trapping Location** | **Sex** | **BCS (score 1-5)** | **Age class^1^** | **Extra postmortem findings** |
| --- | --- | --- | --- | --- | --- |
| M72 | CO | F | 3 | 3 | - |
| M74 | CO | M | 3.5 | 3 | bilateral auxiliary kidney |
| M75 | CO | M | 2.5 | 2.5 | bilateral swelling of inguinal subdermal lymph nodes |
| M76 | CO | M | 3 | 3 | - |
| M20 | FB | M | 3 | 2 | - |
| M21 | FB | M | 3 | 3 | - |
| M22 | FB | F | 2 | 4 | ­­- |
| M25 | FB | F | 2 | 3 | - |
| M35 | FB | M | 3.5 | 2 | - |
| M50 | FB | M | 3.5 | 3 | - |
| M51 | FB | F | 2.5 | 3 | - |
| M52 | FB | F | 2.5 | 2 | - |
| M53 | FB | F | 2 | 1 | - |
| M55 | FB | M | 4 | 3 | - |
| M56 | FB | F | 2 | 2 | - |
| M23 | HB | M | 2.5 | 2 | - |
| M24 | HB | F | 2 | 2 | - |
| M37 | HB | F | 1.5 | 3 | - |
| M38 | HB | F | 2 | 3 | - |
| M49 | HB | F | 2 | 3 | - |
| M77 | IN | F | 3 | 3 | lots of peritoneal fat, lactating |
| M78 | IN | M | 2.5 | 2.5 | - |
| M80 | IN | F | 2 | 2 | - |
| M81 | IN | F | 3 | 3 | pregnant |
| M68 | KB | M | 2.5 | 2.5 | - |
| M69 | KB | M | 2 | 3 | - |
| M70 | KB | M | 2.5 | 3 | bilateral subdermal mass in inguinal region. swollen mesenteric lymph nodes and colon, enteritis |
| M29 | PB | F | 2.5 | 2 | - |
| M30 | PB | M | 2.5 | 2 | - |
| M26 | PH | M | 2.5 | 3 | - |
| M27 | PH | M | 2.5 | 2 | - |
| M28 | PH | M | 3 | 2 | - |
| M31 | PH | M | 3 | 3 | - |
| M32 | PH | M | 3 | 3 | - |
| M33 | PH | M | 3.5 | 3 | - |
| M39 | PH | F | 3 | 2 | - |
| M40 | PH | F | 2.5 | 3 | - |
| M41 | PH | F | not registered | 1 | juveniles with orange eyes |
| M42 | PH | F | not registered | 1 | juveniles with orange eyes |
| M43 | PH | M | 2 | 3 | - |
| M44 | PH | F | 2 | 4 | - |
| M45 | PH | M | 2.5 | 2.5 | - |
| M46 | PH | M | 3 | 3 | - |
| M47 | PH | F | 2 | 3 | yellow liver and left mammary mass |
| M48 | PH | M | 3 | 3 | - |
| M71 | QA | F | 2 | 2.5 | - |
| M73 | QA | M | 3.5 | 3 | - |
| M82 | QA | M | 3 | 3 | - |
| M83 | QA | F | 3 | 3 | pregnant, unilateral multifocal keratopathy |
| M57 | ST | F | 3.5 | 4 | - |
| M58 | ST | F | 2.5 | 3 | - |
| M59 | ST | F | 3 | 3 | mola in uterus |
| M60 | ST | F | 3 | 3 | fluid filled white mass attached to uterine wall |
| M61 | ST | F | 2 | 2 | - |
| M62 | ST | F | 3.5 | 2.5 | cystic masses attached to uterus, broken front canine right |
| M63 | ST | M | 3.5 | 3 | - |
| M64 | ST | F | 3 | 3 | - |
| M65 | ST | F | 2 | 2 | - |
| M66 | ST | M | 3 | 3 | - |
| M67 | ST | F | 3 | 3 | - |

^1^Horst et al., (2001). The Mongoose in the West Indies: the Biogeography and Population Biology of an Introduced Species. In Biogeography of the West Indies: Patterns and Perspectives, 2^nd^ ed.; Woods CA, Sergile FE, eds. CRC Press: Boca Raton, FL, USA, pp. 409-424.
