# Supplementary material for: Unraveling the Gut Microbiome of the Invasive Small Indian Mongoose (Urva auropunctata) in the Caribbean
Source: Microorganisms. 2021 Feb 24;9(3):465. doi: 10.3390/microorganisms9030465 (PMC7996244; doi:10.3390/microorganisms9030465)
Supplement: Supplementary file 1 [file microorganisms-09-00465-s001.zip › Proof_Supplementary Materials_ABecker/Supplementary_Figure3_v2.docx]

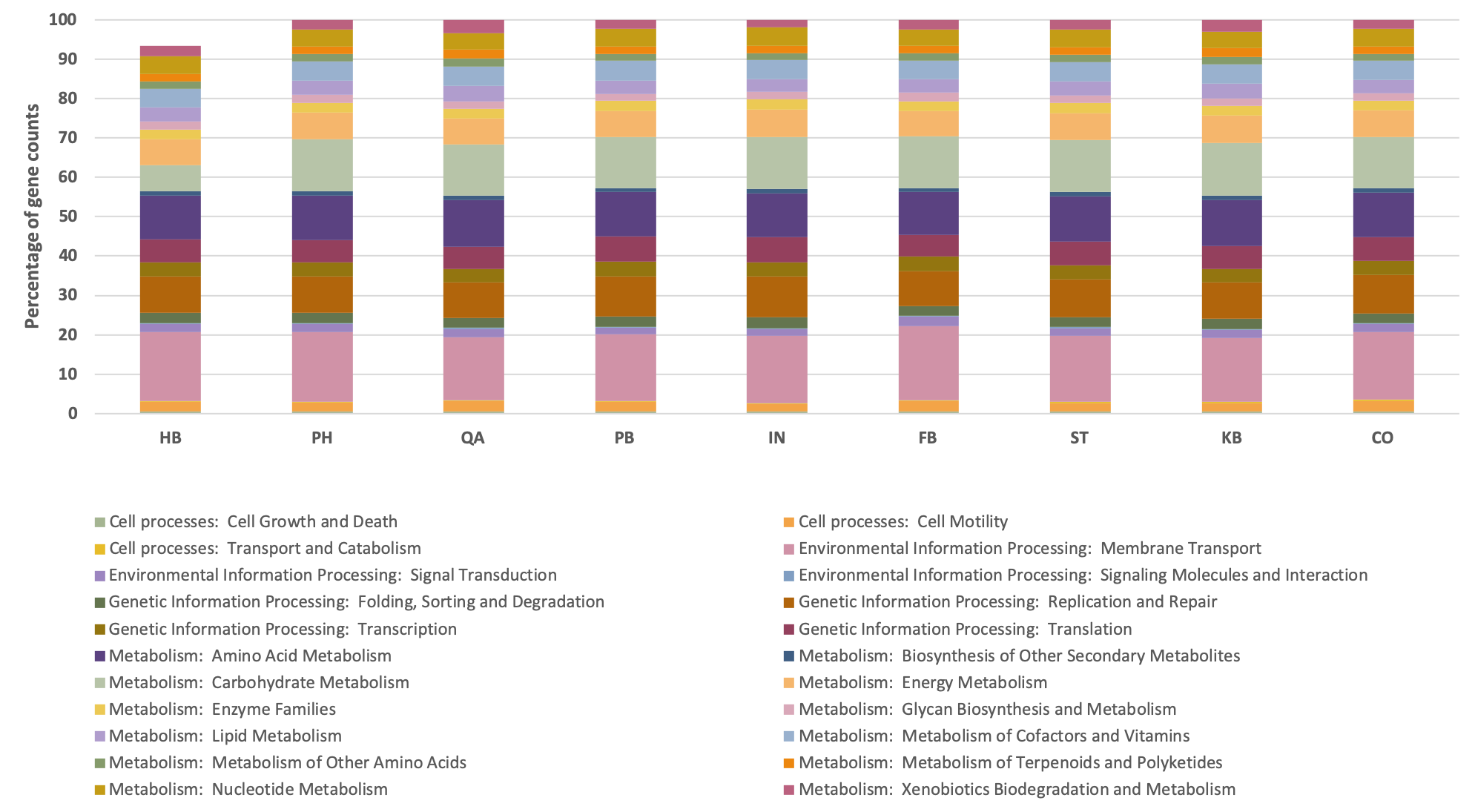


B

A


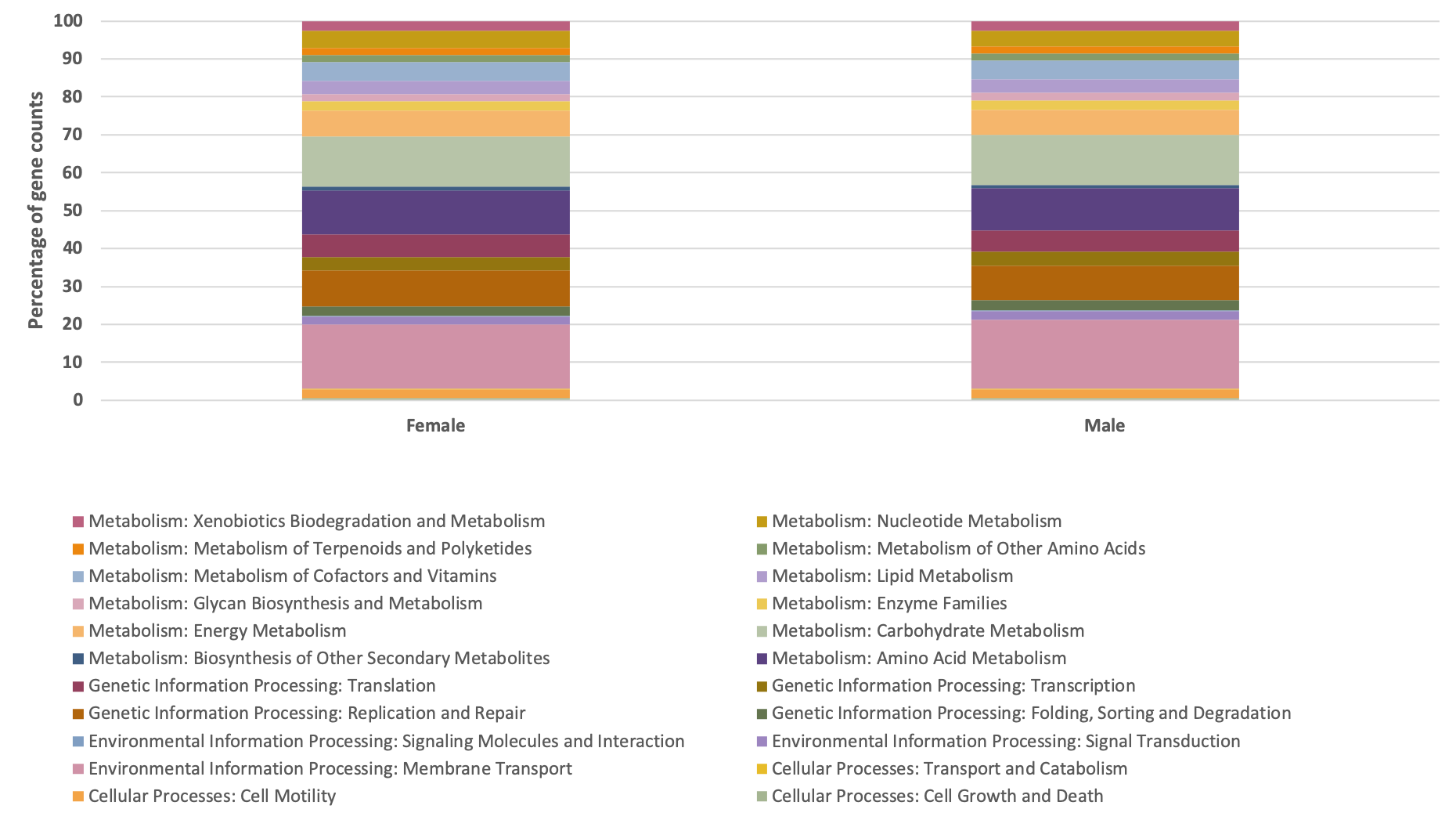


**Supplementary Figure 3.** Percentage of level 2 KO functions among PICRUSt functional predictions per sampling site (A) and sex (B). Functional categories for organismal systems and human diseases were omitted.
