# Supplementary material for: Unraveling the Gut Microbiome of the Invasive Small Indian Mongoose (Urva auropunctata) in the Caribbean
Source: Microorganisms. 2021 Feb 24;9(3):465. doi: 10.3390/microorganisms9030465 (PMC7996244; doi:10.3390/microorganisms9030465)
Supplement: Supplementary file 1 [file microorganisms-09-00465-s001.zip › Proof_Supplementary Materials_ABecker/Supplementary_Table2_proof.docx]

**Supplementary Table 2.** Study trapping locations for free-roaming wild populations of small Indian mongooses (*Urva auropunctata*) on the Caribbean island St Kitts as defined by land cover and forest formation.

| Site Name | Site description | Parish^1^ | Land cover and forest formation^2^ |
| --- | --- | --- | --- |
| CO | Conaree area | St Peter Basseterre | Low density built up land, permanent water, grassy areas and sugar cane |
| FB | Frigate Bay area | St George Basseterre | High-medium density urban or built-up land, golf court, |
| HB | Halfmoon Bay area | St Peter Basseterre | Low density built up land, drought deciduous scrubs, grassy areas |
| IN | Peninsula area | St George Basseterre | Coastal sand, evergreen coastal and mixed forest or shrubland, with or without succulents, emergent wetland and mangrove |
| KB | Key’s beach | St Mary Cayon | Coastal sand |
| PB | Potato Bay area | St George Basseterre | High-medium density urban or built-up land |
| PH | Paradise Heights | St Peter Basseterre | Sugar cane and minor crops, drought deciduous open woodland and scrubland and protected National Park Royal Basseterre Valley Aquifer |
| QA | Quarry | St Peter Basseterre | Actively exploited quarry |
| ST | Strip area | St George Basseterre | Coastal sand, evergreen coastal and mixed shrubland, with or without succulents |

^1^Parishes are administrative divisions of the federation of St Kitts and Nevis. ^2^Description of trapping locations based on Helmer et al. (2008). Distributions of land cover and forest formations for St Kitts, Nevis, St Eustatius, Grenada and Barbados from satellite imagery. Caribb J Sci. 2008;44:175-98. ^3^Distribution pattern of mongooses in the wider Carribean as described by Louppe et al. (2020). The Globally invasive small Indian mongoose *Urva auropunctata* is likely to spread with climate change. Scientific Reports, 2020;10:1-11.
