# Supplementary material for: Unraveling the Gut Microbiome of the Invasive Small Indian Mongoose (Urva auropunctata) in the Caribbean
Source: Microorganisms. 2021 Feb 24;9(3):465. doi: 10.3390/microorganisms9030465 (PMC7996244; doi:10.3390/microorganisms9030465)
Supplement: Supplementary file 1 [file microorganisms-09-00465-s001.zip › Proof_Supplementary Materials_ABecker/Supplementary_Figure2_v1.docx]

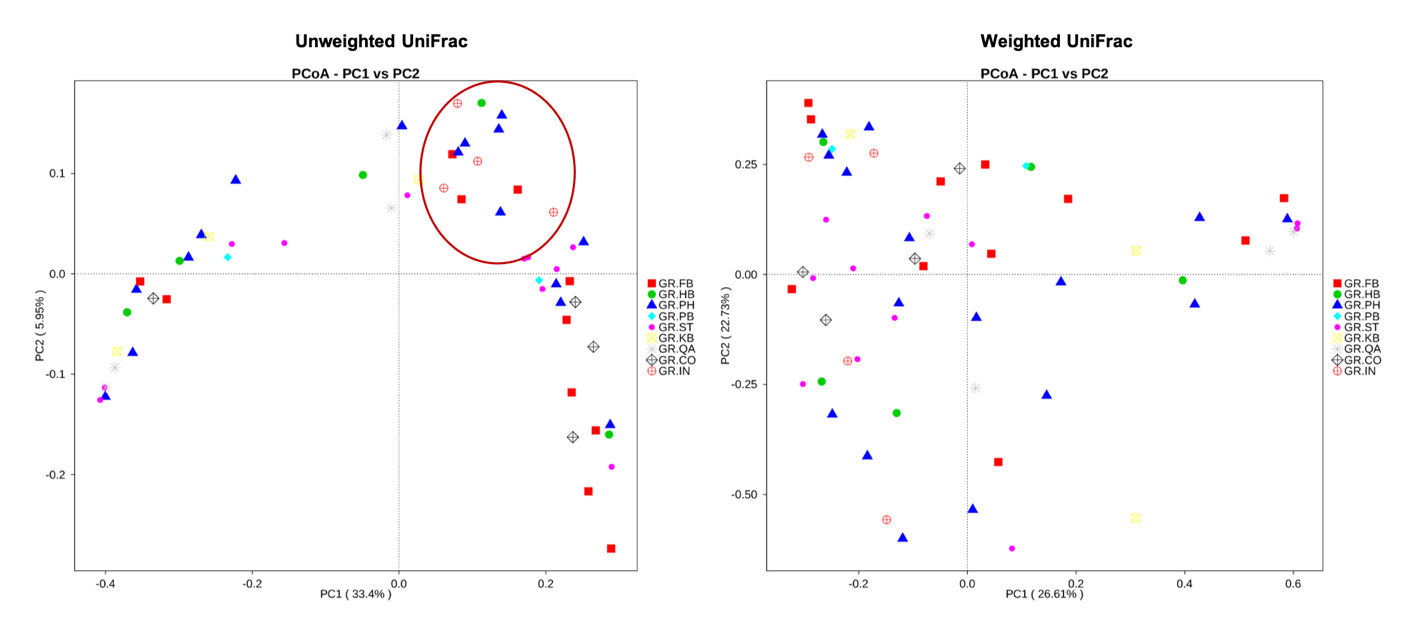


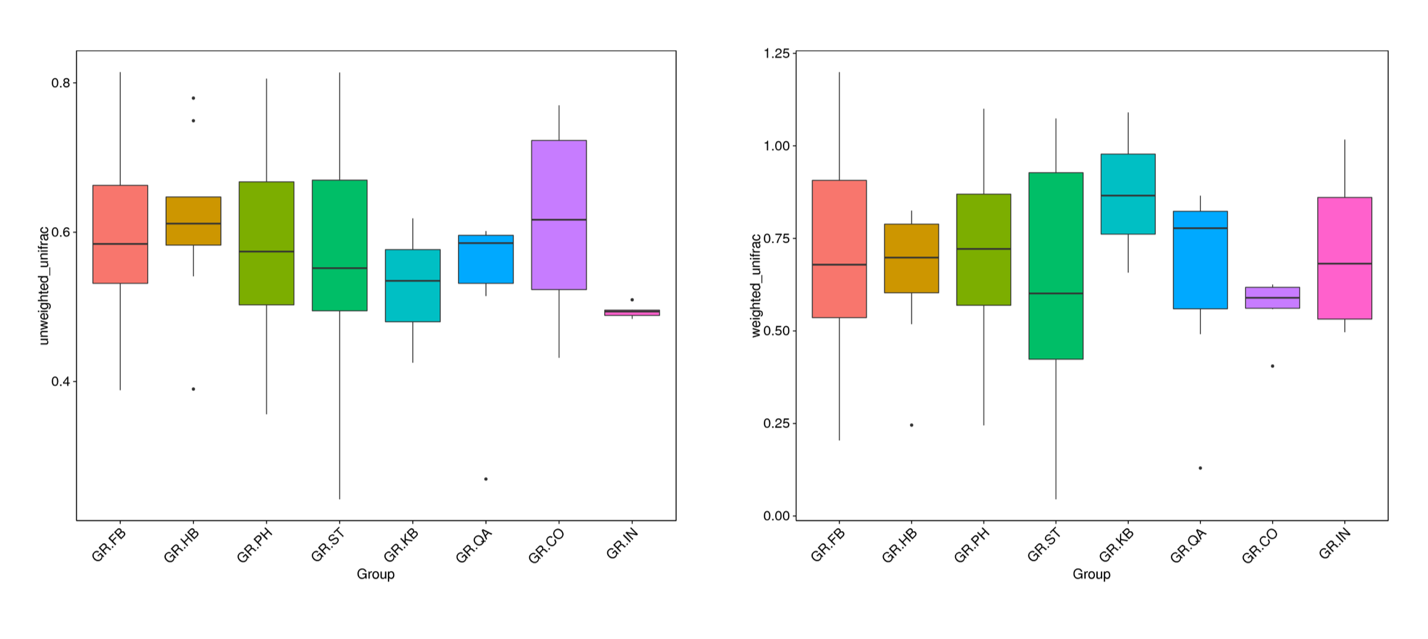


**Supplementary Figure 2.** **(A)** Principal component analysis (PCoA) of the gut microbiota of fecal samples from 60 small Indian mongooses trapped at nine different locations, based on unweighted and weighted UniFrac distances. Circle shows cluster of samples collected at the peninsula (GR.IN). **(B)** Boxplots of unweighted and weighted UniFrac distances, with significant difference in unweighted UniFrac between samples collected at the peninsula and five other locations (GR.IN vs GR.CO, *P*= 0.023; GR.IN vs. GR. FB, *P*= 0,003; GR.IN vs GR.HB, *P*= 0.005, GR.IN vs GR.PH, *P*= 0.010; GR.IN vs. GR.ST, *P*= 0.021).
