# Supplementary material for: Unraveling the Gut Microbiome of the Invasive Small Indian Mongoose (Urva auropunctata) in the Caribbean
Source: Microorganisms. 2021 Feb 24;9(3):465. doi: 10.3390/microorganisms9030465 (PMC7996244; doi:10.3390/microorganisms9030465)
Supplement: Supplementary file 1 [file microorganisms-09-00465-s001.zip › Proof_Supplementary Materials_ABecker/Supplementary_Figure4_v2.docx]

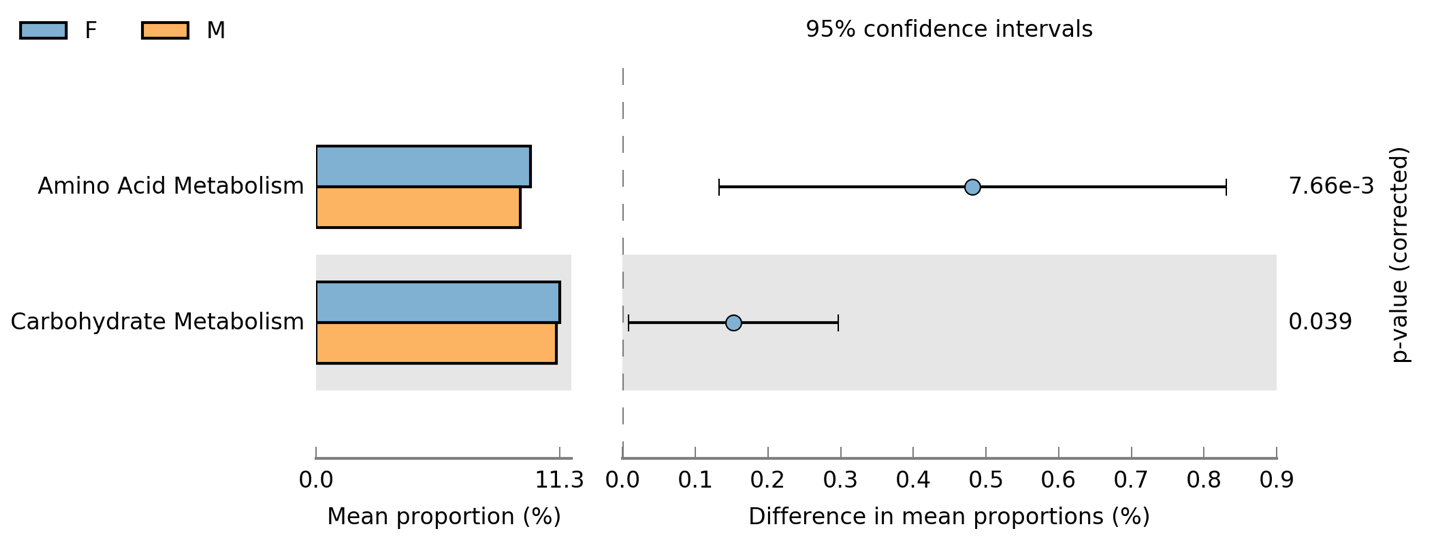


**Supplementary Figure 4.** Extended error bar plots show significant differences between mean proportions of functional predictions at KEGG level 2 “Amino acid metabolism” and “Carbohydrate metabolism” (based on two-sided Welch’s t-test).
