# Supplementary material for: Unraveling the Gut Microbiome of the Invasive Small Indian Mongoose (Urva auropunctata) in the Caribbean
Source: Microorganisms. 2021 Feb 24;9(3):465. doi: 10.3390/microorganisms9030465 (PMC7996244; doi:10.3390/microorganisms9030465)
Supplement: Supplementary file 1 [file microorganisms-09-00465-s001.zip › Proof_Supplementary Materials_ABecker/Supplementary_Figure1_v1.docx]

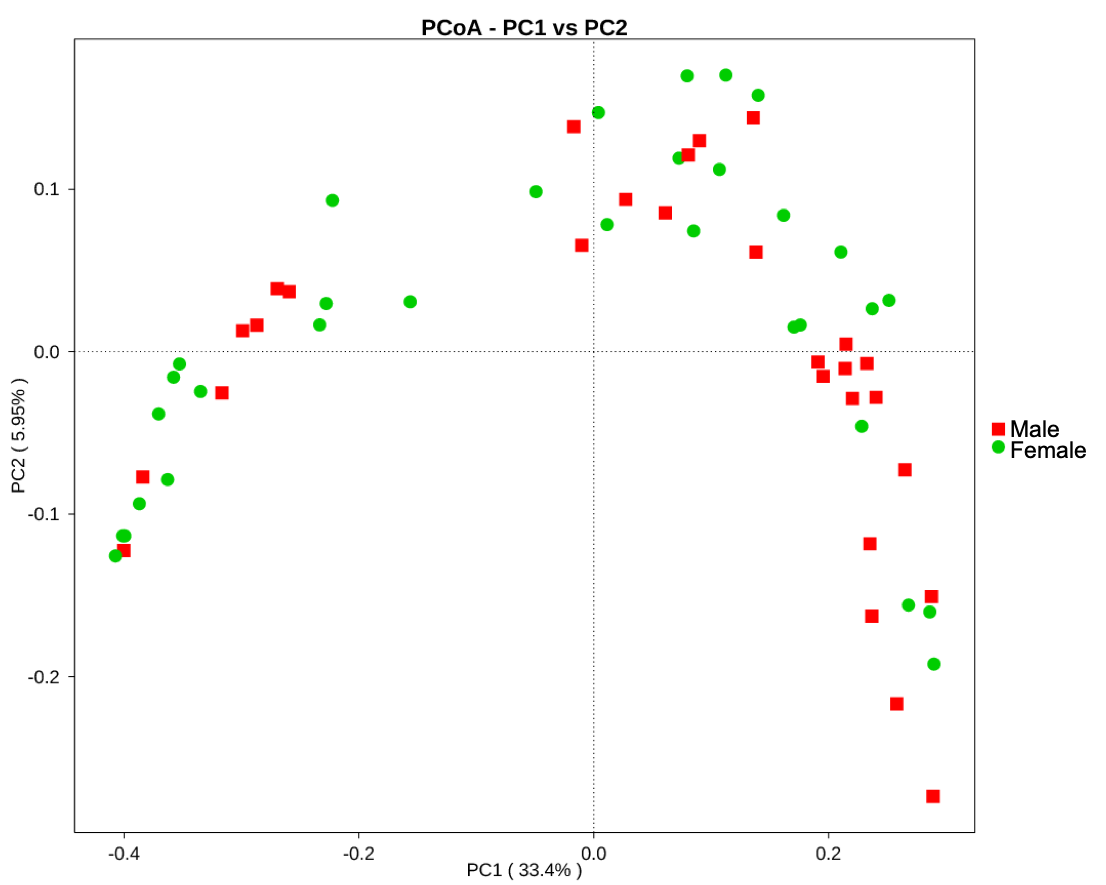


**Supplementary Figure 1.** Principal component analysis (PCoA) of the gut microbiota of male (red, n=28) and female (green, n=32) small Indian mongooses, based on unweighted UniFrac distances.
